# Computational assessment of the primary and secondary antioxidant potential of alkylresorcinols in physiological media†

**DOI:** 10.1039/d3ra05967g

**Published:** 2023-10-09

**Authors:** Houssem Boulebd, Maciej Spiegel

**Affiliations:** a Department of Chemistry, Faculty of Exact Science, University of Constantine 1 Constantine 25000 Algeria boulebd.houssem@umc.edu.dz; b Department of Pharmacognosy and Herbal Medicines, Faculty of Pharmacy, Wroclaw Medical University Borowska 211A 50-556 Wroclaw Poland

## Abstract

Alkylresorcinols are a group of natural phenolic compounds found in various foods such as whole grain cereals, bread, and certain fruits. They are known for their beneficial health effects, such as anti-inflammatory and anti-cancer properties. This study aimed to evaluate the antioxidant activity of two typical alkylresorcinols namely olivetol and olivetolic acid (Oli and OliA) under physiological conditions. The free radical scavenging capacity of Oli and OliA toward oxygenated free radicals (HO˙ and HOO˙ radicals) was investigated using thermodynamic and kinetic calculations. The results revealed that Oli and OliA are potent scavengers of HO˙ radical in both polar and lipid media, acting exclusively *via* the FHT (formal hydrogen transfer) mechanism. Moreover, they demonstrated excellent scavenging activity toward HOO˙ radical in water *via* the SET (single electron transfer) mechanism, outperforming the common antioxidant BHT. In lipid media, Oli and OliA showed moderate scavenging activity toward HOO˙ radical *via* the FHT mechanism. Significant prooxidant potential of OliA^−^ was also demonstrated through the formation of complexes with copper ions. Additionally, docking studies indicate that the compounds exhibited a good affinity for ROS-producing enzymes, including myeloperoxidase (MP), cytochrome P450 (CP450), lipoxygenase (LOX), and xanthine oxidase (XO), highlighting their potential as natural antioxidants with promising therapeutic applications.

## Abstract 2

Olivetol and olivetolic acid exhibit significant antioxidant potential in physiological polar environments, acting as free radical scavengers, metal ion chelators, and inhibitors of reactive oxygen species (ROS)-producing enzymes.

## Graphical abstract

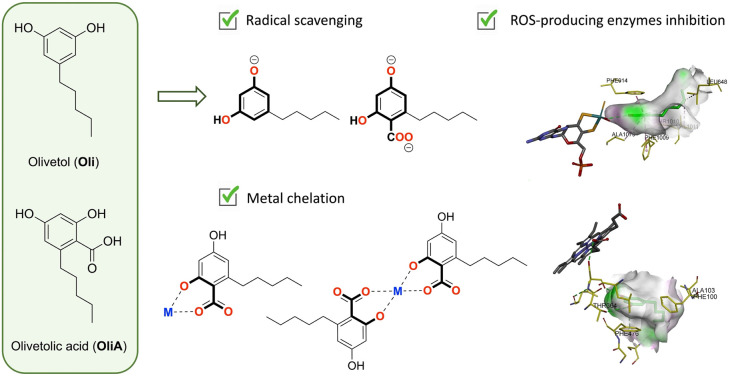

## 1. Introduction

Reactive oxygen species (ROS) are highly reactive molecules generated as by-products of normal cellular metabolism and environmental factors such as UV radiation, cigarette smoke, and pollution. These ROS, if not controlled by the endogenous antioxidant system, can cause damage to cell structures such as DNA, lipids, and proteins, leading to oxidative stress (OS). OS is linked to a variety of human diseases such as cancer, cardiovascular disease, diabetes, and neurodegenerative disorders.^1–3^ Antioxidants are natural or synthetic compounds that protect cells from excessive ROS production.^4^ They can be classified into primary and secondary antioxidants, based on their mechanism of action.^5^ Primary antioxidants scavenge free radicals by donating a hydrogen atom or electron to neutralize the reactive species. Examples of primary antioxidants include vitamins C and E, carotenoids, and glutathione.^6^ Secondary antioxidants can prevent oxidative damage through various mechanisms, including chelation of metal ions and inhibition of ROS-producing enzymes.^7^ Metal ions, such as iron and copper, can promote oxidation by catalyzing the formation of free radicals, but secondary antioxidants can sequester these metal ions through chelation, thereby inhibiting their pro-oxidant activity. In addition, some secondary antioxidants can directly inhibit enzymes that generate ROS, such as lipoxygenases and xanthine oxidase, which can lead to a reduction in OS and damage.^8^ The combination of primary and secondary antioxidants provides a synergistic effect that enhances the overall antioxidant capacity of the system. Understanding the different mechanisms of action of primary and secondary antioxidants is crucial in developing strategies to prevent or treat OS-related diseases.

Alkylresorcinols (ARs) are a group of natural phenols found mainly in the outer layers of grains such as wheat, rye, and barley.^9^ They are characterized by a long hydrophobic alkyl chain and a hydrophilic resorcinol ring. ARs have been studied extensively for their potential health benefits, including anti-inflammatory, anti-tumor, and antimicrobial properties.^10^ Some studies have also suggested that they may help regulate blood lipid levels and protect against cardiovascular disease.^11,12^ ARs have also been used as biomarkers of whole-grain intake in dietary studies.^13^ ARs can serve as a treatment for type 2 diabetes by enhancing the body's sensitivity to insulin.^14^ The studies on ARs have shown their significant antioxidant activity, making them a promising natural ingredient for the food industry. Elder A. S. *et al.* highlighted that the antioxidant effectiveness of ARs varies depending on the food system. In bulk oils, their antioxidant activity decreases with an increase in alkyl chain length, while in oil-in-water emulsions, the optimum antioxidant activity is observed at intermediate alkyl chain length (C21:0). ARs were found to scavenge radicals effectively in oils, while physicochemical phenomena, such as partitioning, drive their antioxidant activity in emulsions.^15^ Similarly, the same research group explored the antioxidant activity of rye bran extract containing ARs in an oil-in-water emulsion. The extract demonstrated the ability to inhibit lipid oxidation reactions in the emulsion. The partitioning behavior of ARs in the continuous phase of the emulsion contributed to their radical scavenging antioxidant effect, as they associated with the lipid phase and surfactant micelles.^16^ Furthermore, investigations on rye samples revealed that the antioxidant activity of ARs decreased from the outermost to innermost fractions of the bran. The amount of ARs in bran fractions positively correlated with their antioxidant protection against free radical damage.^17^ Additionally ARs from rye bran and other whole-grain cereal products exhibited significant radical-scavenging activity, with no major impact of alkyl chain length on their antioxidant properties.^18^ Finally, the study on the bryozoan *Schizomavella mamillata* isolated six new ARs with radical scavenging activity higher than the Trolox standard in the ABTS antioxidant assay.^19^ Overall, the findings from these studies emphasize the potential importance of ARs as natural antioxidants for the food industry, particularly for protecting food products during processing and storage. Their ability to scavenge radicals and inhibit lipid peroxidation makes them valuable candidates for replacing synthetic antioxidants with naturally derived alternatives. Despite the notable antioxidant potency of ARs, little research has been conducted to investigate their mechanism of action against free radicals and their secondary antioxidant effects.

**Fig. 1:**
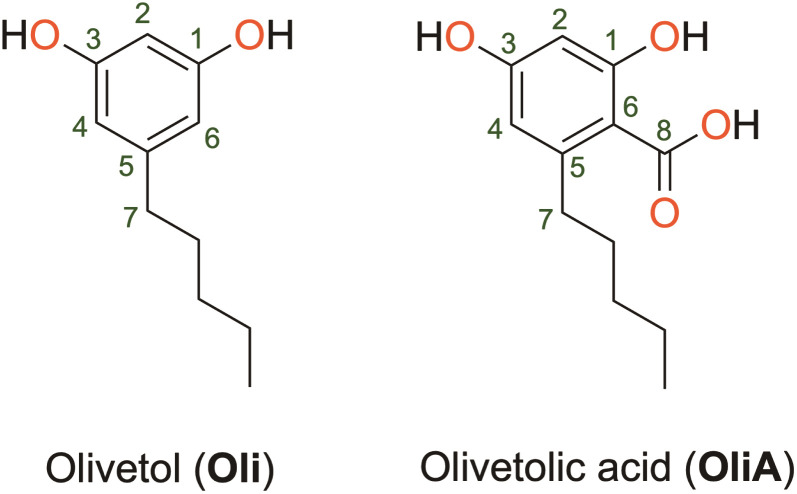
Molecular structure of olivetol and olivetolic acid.

Olivetol (Oli) and olivetolic acid (OliA) shown in Fig. 1 are two derivatives of ARs that have gained attention for their potential medicinal uses. Oli has been found to have various pharmacological effects, including analgesic and anti-inflammatory properties.^20^ It is also a precursor for the synthesis of cannabigerol, a non-psychoactive cannabinoid found in *Cannabis* plants.^21^OliA, on the other hand, is a polyketide compound that differs from Oli by the presence of a carboxylic acid substituent. It is a precursor to tetrahydrocannabinol, the main psychoactive compound found in *Cannabis* plants.^22^ It has been found to have antifungal and antibacterial properties, as well as potential anti-inflammatory and anti-cancer effects.^23,24^ However, despite their promising biological properties, limited studies have been conducted on their antioxidant capacity. This is a significant limitation, as these compounds have the potential to be used as natural alternatives to toxic synthetic compounds like BHT and BHA, which are commonly used as preservatives in the food industry.^25^

The aim of this study was to investigate the antioxidant activity and mechanism of Oli and OliA as typical alkylresorcinols derivatives. Firstly, the free radical scavenging activity of Oli and OliA was evaluated towards oxygenated free radicals (HO˙ and HOO˙) using thermodynamic and kinetic calculations based on the DFT method. The study examined all possible reaction pathways with HO˙ and HOO˙ while considering the influence of pH and physiological conditions. The results were compared with those of the commonly used antioxidant BHT, as well as with available experimental data. Furthermore, the pro-oxidant behavior of OliA was studied with respect to Fe^3+^ and Cu^2+^ ions in aqueous media. Finally, molecular docking studies were carried out to estimate the affinity of Oli and OliA towards ROS-producing enzymes, including myeloperoxidase (MP), cytochrome P450 (CP450), lipoxygenase (LOX), and xanthine oxidase (XO). This research sheds light on the antioxidant properties of alkylresorcinols and their potential as natural alternatives to synthetic antioxidants.

## 2. Computation procedures

### 2.1. Quantum chemistry calculations

There are three distinct processes through which phenolic compounds exhibit their antiradical action, namely FHT (formal hydrogen transfer), RAF (radical adduct formation), and SET (single electron transfer).^26–30^ FHT is a one-step process where the transfer of a hydrogen atom from the antioxidant to the free radical takes place. This mechanism can occur in both polar and lipid environments, and it can be described using the BDE of the active OH/CH bonds of the antioxidant. On the other hand, RAF is a process where free radicals are combined with antioxidants, leading to the formation of the product [antioxidant-free radical]˙ in one step.^31^ The Gibbs free energy of the reaction is used to describe this mechanism. Unlike FHT and RAF, SET involves an electron transfer and can only occur in polar environments.^32^ In these environments, the phenolic OH bonds of the antioxidant can exist in both protonated and deprotonated forms, allowing electron transfer from the neutral or anionic state of the antioxidant. However, SET from an undissociated phenol is often not feasible, making it significant only for deprotonated phenols. The dominant step in this pathway can be described using the Gibbs free energy of electron transfer. The mechanisms mentioned above are described by the following equations:Ar–OH + R˙ → Ar–O˙ + ROH (FHT)Ar–OH + R˙ → [Ar–OH–R]˙ (RAF)Ar–OH → Ar–O^−^ + H^+^; Ar–O^−^ + R˙ → Ar–O˙ + R^−^ (SET)

In this study, all of the aforementioned mechanisms have been examined through thermodynamic and kinetic calculations. To calculate the molecular geometry and frequencies of the molecules involved in these processes, the DFT approach at the M06-2X/6-311++G(d,p) level of theory has been employed. Previous research has validated the reliability of this method.^33^ The number of imaginary frequencies was used to confirm both the ground (0) and transition states (1), which was also confirmed using intrinsic reaction coordinate (IRC) calculations. To simulate physiological conditions in polar and lipid environments, the solvation effect of water and pentyl ethanoate was utilized by employing the SMD solvation model.^34^ The BDE values have been calculated as reported in the literature.^35^ The p*K*_a_ values have been calculated according to the literature using the following equation:^36^AH + OH^−^(3H_2_O) → A^−^(H_2_O) + 3H_2_O
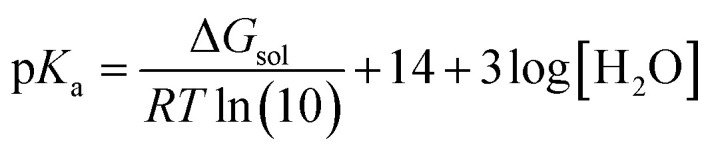
where Δ*G*_sol_ is the Gibbs free energy of the reaction in solution, *R* is the gas constant, and *T* is the temperature (298.15 K). Water is used as a co-reactant in p*K*_a_ calculations because it simplifies the modelling of proton solvation and improves accuracy by taking advantage of water's well-known solvation properties. This approach overcomes the computational difficulties associated with accurately estimating proton solvation energy.

The kinetics of the reactions of Oli and OliA with oxygenated free radicals (HO˙ and HOO˙ radicals) have been modeled following the QM-ORSA protocol.^37,38^ QM-ORSA has been proposed as an accurate methodology for predicting the kinetics of free radical scavenging activity and has been verified against experimental results.^37^ Transition state theory (TST) has been employed to calculate the rate constants (*k*) as shown in the following equation:^39–44^
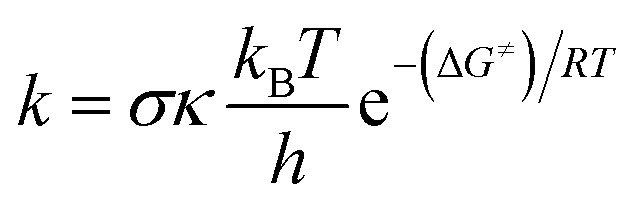
where *σ* is reaction symmetry number, *κ* is tunneling correction, *k*_B_ is Boltzmann constant, ℏ is Planck constant, and Δ*G*^≠^ Gibbs free energy of activation.^45–47^ The tunneling correction was computed using the Eckart barrier.^47^ The apparent rate constants (*k*_app_, rate constants close to the diffusion limit) were corrected using the Collins–Kimball theory.^48^ Branching ratios (*Γ*, %) were calculated as follows:
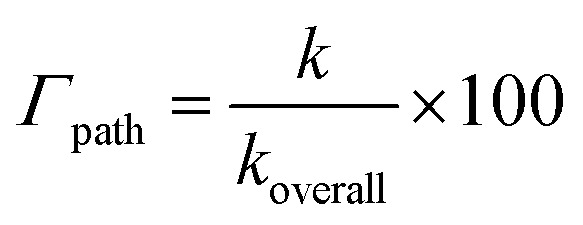
where *k* and *k*_overall_ are the rate constants of a specific reaction path and the sum of the rate constants of all reaction paths, respectively.

Marcus theory has been used to predict the Gibbs free energy of activation of the single electron transfer mechanism:^49^
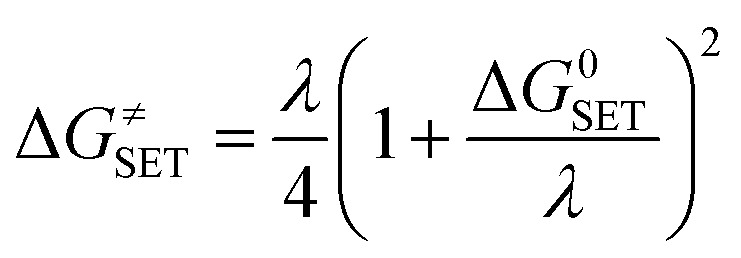
*λ* ≈ Δ*E*_SET_ + Δ*G*^0^_SET_where *λ* is the nuclear reorganization energy, Δ*G*^0^_SET_ is the Gibbs free energy of reaction, and Δ*E*_SET_ is the nonadiabatic energy difference between reactants and vertical products. The DFT calculations were performed with the Gaussian 09 suite of programs.^50^

The complexation process was studied by mean of sequestrating Cu(ii) aqua complexes by the carboxylic oxygen and the adjacent hydroxyl group of OliA, as this is the only viable scaffold capable of efficient chelation. The M06 functional^51^ was used, instead of M06-2X,^52^ since the former was parameterized to include both metallic and non-metallic systems. The Stuttgart/Dresden effective core potentials and the already presented Pople's basis set were used to describe the electron density of the chemically irrelevant core and valence electrons, respectively.^53,54^ Similar methodology has been used in the previous work.^8^ The chelating potential was determined by the mean of Gibbs free energy of complexation 
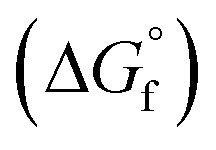
 for a reaction with a general scheme:[M(H_2_O)_6_]^*n*^ + A^*m*^ ⇄ [M(H_2_O)_4_A]^*n*−*m*^ + 2H_2_Owhere M denotes the metal, *n* its charge, A describes the antioxidant, and *m* the charge of the antioxidant species. The apparent equilibrium constants (*K*^app^_i_) were then calculated according to the set of formulas:
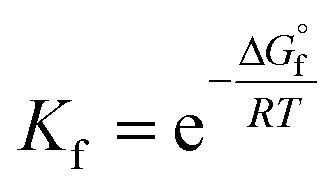
*K*^II^_i_ = *K*_f_ × ^m^*f*_i_*K*^app^_i_ = ∑*K*^II^_i_where *K*_f_ is the equilibrium constant at the studied complexation site of a given species, *K*^II^_i_ equals the equilibrium constant corrected by the molar fraction, ^m^*f*_i_, of the considered i species under the studied conditions.

Although the resulting complexes are generally considered less reactive, they may in fact exhibit pro-oxidative potential, causing depletion of internal antioxidants or generation of new radicals, and consequently facilitating further oxidative damage. This behavior was assessed by examining the thermochemistry and kinetics of the single-electron transfer reactions between them and biologically relevant reducing agents, such as ascorbate ion and superoxide anion radical:[M(H_2_O)_4_A]^*n*^ + Asc^−^/O_2_˙^−^ → [M(H_2_O)_4_A]^*n*−1^ + Asc˙/O_2_

### 2.2. Docking studies

The optimized structures of Oli and OliA obtained by DFT calculations has been employed for the docking study. The Protein Data Bank (PDB) (https://www.rcsb.org) was used to get the coordinates for myeloperoxidase (MP, PDB: 5WDJ), cytochrome P450 (CP450, PDB: 1OG5), lipoxygenase (LOX, PDB: 1JNQ), and xanthine oxidase (XO, PDB: 3NVY). The proteins were cleansed of the ligand, all water atoms, heteroatoms, and co-crystallized solvent. Using AutoDockTools (v. 1.5.6), partial charges and hydrogens were added to the protein and ligand. A 25-point grid, spaced one unit apart, has been used to construct the search space, which is centered at the protein's catalytic center. AutoDock Vina (v. 1.1.2) was used for the whole docking investigation.^55^ All the parameters have been set to their default settings, with the exception of num modes, which is set to 10. The BIOVIA Discovery Studio (https://3dsbiovia.com/) was used to draw the figures.

## 3. Results and discussion

### 3.1. Thermodynamic evaluation of the reactivity towards oxygenated free radicals in the gas phase

**Fig. 2:**
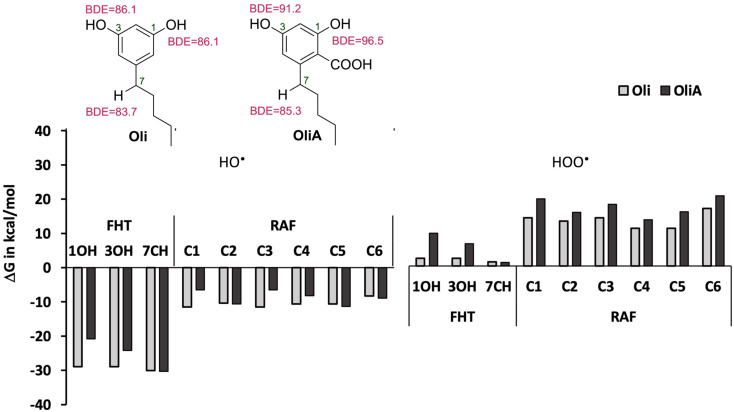
Reactivity of Oli and OliA towards oxygenated free radicals following the possible antioxidant mechanisms in the gas phase.

As a first step, the reactivity of Oli and OliA with oxygenated free radicals was assessed in the gas phase in all conceivable positions using thermodynamic calculations. The Gibbs free energies of reactions as well as the BDE values of the active bonds are shown in Fig. 2 and Table S1 in ESI.† It was found that the reaction of both compounds with the HO˙ radical is exergonic in all positions with Δ*G*_s_ ranging from −6.5 to −30.3 kcal mol^−1^, indicating that the HO˙ radical is non-selective and may attack in all positions. In contrast, the reaction with the HOO˙ radical is less energetically favorable and seems to be possible only at the 7CH group for both compounds and at the OH groups for Oli. Therefore, only energetically favorable reactions are taken into account in the kinetic study. In terms of BDE values, the 7CH bonds display the lowest values of 83.7 and 85.3 kcal mol^−1^ for Oli and OliA respectively, suggesting that they are the most thermodynamically susceptible bonds to break. Compared with BHT (calculated BDE = 78.10 kcal mol^−1^),^56^ these values are approximately 5 to 7 kcal mol^−1^ higher, indicating that BHT may be a more effective antioxidant through hydrogen atom transfer than Oli and OliA.

### 3.2. Kinetic behavior in physiological environments

#### 3.2.1. Acid–base equilibrium at physiological pH

**Fig. 3:**
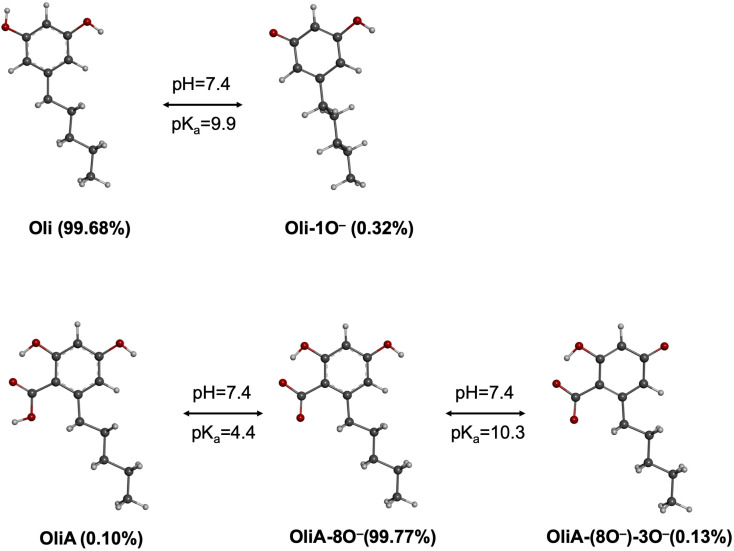
Deprotonated states of Oli and OliA at physiological pH.

Before starting the kinetic study under physiological conditions, it is necessary to examine the behavior of the different OH groups at physiological pH. For this purpose, the p*K*_a_ values of Oli and OliA were calculated following a protocol described in the literature, and the results obtained are shown in Fig. 3.^36^Oli has a p*K*_a_ value of 9.9 which means that, at physiological pH, the molecule exists predominantly in the neural form (99.68%) with the presence of a minimum amount of the deprotonated form (0.32%). On the other hand, OliA has two p*K*_a_ values of 4.4 and 10.3. These values show that the mono-anionic form of OliA dominates at physiological pH (99.77%) with the presence of a very small amount of both the neutral and dianionic forms (<0.13%). Although the low concentration of the mono-anionic form of Oli and the neutral and dianionic forms of OliA should not impact the reactivity of the molecules through hydrogen transfer or radical adduct formation mechanisms, the dissociated form, even in small amounts, can significantly affect the reactivity through the electron transfer mechanism.^57^ Therefore, only the dominant form was considered for the FHT and RAF mechanisms, while all species present at physiological pH were examined for the SET mechanism. Concerning the lipid medium, the dissociation of the HO bond is not possible and thus only the neutral form is present under these conditions.

#### 3.2.2. Kinetics of the hydroxyl radical scavenging under physiological conditions

**Fig. 4:**
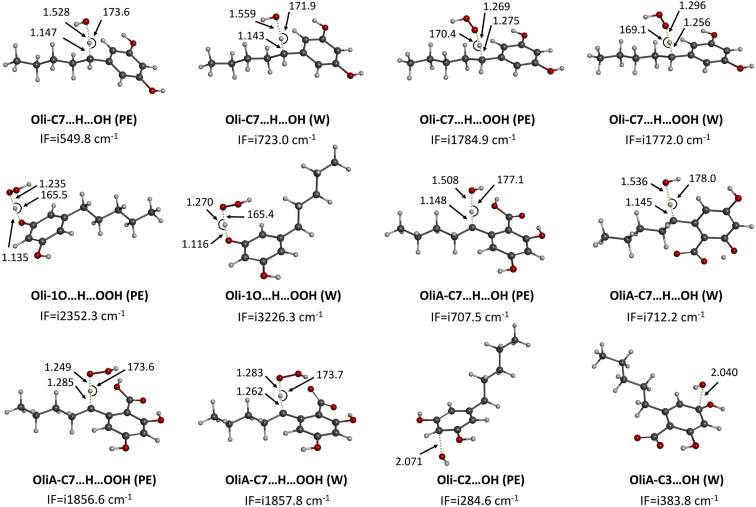
Localized TSs of the reaction of Oli and OliA with HO˙ and HOO˙ under physiological conditions. IF = imaginary frequencies, W = water, and PE = pentylethanoate.

The findings of the kinetic study of the reaction of Oli and OliA with the HO˙ radical under physiological conditions are shown in Table 1 and the localized transition states are illustrated in Fig. 4. Analysis of the data in Table 1 indicates that Oli and OliA react with the HO˙ radical at almost the same rate constant under both conditions studied (*k*_overall_ of the order of 10^9^ M^−1^ s^−1^). Compared to the reference antioxidant, BHT, this value is somewhat lower, which means that BHT is more reactive than Oli and OliA toward the HO˙ radical under physiological conditions. The analysis of the individual rate constants shows that the FHT mechanism at the OH group is almost exclusive for both compounds in both environments (*Γ* ≈ 100%). It is important to note that the latter reaction is characterized by a rate constant equal to the diffusion limit, which is also the case of other antioxidants such as viniferifuran,^58^ daphnetin,^59^ and resveratrol.^60^ In comparison to recognized antioxidants such as caffeine (*k*_overall_ = 2.15 × 10^9^ M^−1^ s^−1^ in water)^61^ and glutathione (*k*_overall_ = 7.68 × 10^9^ M^−1^ s^−1^ in water),^62^Oli and OliA demonstrates high HO˙ scavenging activity in physiological media.

**Table 1:** Calculated Δ*G*^≠^ (kcal mol^−1^), *κ*, *k* (M^−1^ s^−1^), and *Γ* (%) of the reaction of Oli and OliA with HO˙ radical in water (W) and pentylethanoate (PE)

Comp.	Sol.	Mechanism		State	Δ*G*^≠^	*κ*	*k*_app_	*f* a	*k*_f_ b	*Γ*	*k*_overall_
Oli	PE	FHT	1OH	Oli	—	—	2.60 × 10^9^^d^	—	—	48	5.42 × 10^9^
			3OH		—	—	2.60 × 10^9^^d^			48	
			7CH		6.0	0.6	1.60 × 10^8^			3	
		RAF	C1–C6^c^		—	—	5.81 × 10^7^			1	
	W	SET		Oli	—	—	—	0.9968	∼0	0	4.73 × 10^9^
				Oli^−^	0.1	20.2^e^	2.10 × 10^9^	0.0032	6.72 × 10^6^	0	
		FHT	1OH	Oli	—	—	2.30 × 10^9^^d^	0.9968	2.29 × 10^9^	48	
			3OH		—	—	2.30 × 10^9^^d^		2.29 × 10^9^	48	
			7CH		4.3	0.0	9.20 × 10^7^		9.17 × 10^7^	2	
		RAF	C1–C6^d^		—	—	—		4.48 × 10^7^	1	
OliA	PE	FHT	1OH	OliA	—	—	2.60 × 10^9^^d^	—	—	49	5.36 × 10^9^
			3OH		—	—	2.60 × 10^9^^d^			49	
			7CH		6.1	0.3	5.40 × 10^7^			1	
		RAF	C1–C6^d^		—	—	1.04 × 10^8^			2	
	W	FHT	1OH	OliA^−^	—	—	2.30 × 10^9^^d^	0.9977	2.29 × 10^9^	49	4.70 × 10^9^
			3OH		—	—	2.30 × 10^9^^d^		2.29 × 10^9^	49	
			7CH		4.5	0.0	9.1 × 10^7^		9.08 × 10^7^	2	
		SET		OliA	—	—	—	0.0010	∼0	0	
				OliA^−^	—	—	—	0.9977	∼0	0	
				OliA^−2^	0.2	21.2^e^	1.50 × 10^9^	0.0013	1.95 × 10^6^	0	
		RAF	C1–C6^d^	OliA^−^	—	—	—	0.9977	1.65 × 10^7^	0	
BHT^63^	PE	—									1.13 × 10^11^
	W	—									1.39 × 10^12^

a Mole fraction.

b *k*_f_ = *f* × *k*_app_.

c ∑*k*_RAF_ (Table S2 in ESI).

d Diffusion constant.

e The nuclear reorganization energy (*λ*).

#### 3.2.3. Kinetics of the hydroperoxyl radical scavenging under physiological conditions

The findings of the kinetic study of the reaction of Oli and OliA with the HOO˙ radical under physiological conditions are shown in Table 2 and the localized transition states are illustrated in Fig. 4. Unlike the reactivity towards the HO˙ radical, the reactivity towards the HOO˙ radical appears to be more important in water than in pentyl ethanoate for both compounds. The rate constant of Oli is about 10^5^ times greater in water than in pentyl ethanoate while that of OliA is about 10^6^ times greater. This difference in reactivity between the two environments is due to the involvement of the SET mechanism possible only in water and contributes 100% to the overall rate constant. In comparison, BHT is significantly more reactive than Oli and OliA in the lipid medium (1.70 × 10^4^ M^−1^ s^−1^*vs.* 1.92 × 10^1^ and 8.70 × 10^−2^ M^−1^ s^−1^), while its reactivity is less important in the polar medium (2.51 × 10^5^ M^−1^ s^−1^*vs.* 5.76 × 10^6^ and 1.87 × 10^6^ M^−1^ s^−1^). Oli and OliA are about 10 times more active than BHT, which suggests that these molecules are powerful antioxidants in water at physiological pH. On the other hand, Oli appears to be more reactive than OliA in water with a threefold higher rate constant. Nevertheless, this dissimilarity in reactivity is not noteworthy, implying that the existence of the carboxyl group does not influence the molecule's reactivity. In comparison with other antioxidants such as Trolox (*k*_overall_ = 8.96 × 10^4^ M^−1^ s^−1^),^64^ guaiacol (*k*_overall_ = 2.83 × 10^6^ M^−1^ s^−1^),^65^ eugenol (*k*_overall_ = 1.55 × 10^6^ M^−1^ s^−1^),^65^ carnosic acid (*k*_overall_ = 4.73 × 10^6^ M^−1^ s^−1^),^66^ cannabidiol (*k*_overall_ = 9.09 × 10^6^ M^−1^ s^−1^),^67^ and cannabidiolic acid (*k*_overall_ = 1.30 × 10^6^ M^−1^ s^−1^),^68^ it can be concluded that Oli and OliA are potent antioxidants in water.

**Table 2:** Calculated Δ*G*^≠^ (kcal mol^−1^), *κ*, *k* (M^−1^ s^−1^), and *Γ* (%) of the reaction of Oli and OliA with HOO˙ radical under physiological conditions

Comp.	Sol.	Mechanism		State	Δ*G*^≠^	*κ*	*k*_app_	*f* a	*k*_f_ b	*Γ*	*k*_overall_
Oli	PE	FHT	1OH	Oli	19.1	145.8	9.60 × 10^0^	—	—	50	1.92 × 10^1^
			3OH		19.1	145.8	9.60 × 10^0^			50	
			7CH		22.1	55.2	2.30 × 10^−2^			0	
	W	SET		Oli	—	—	—	0.9968	∼0	0	5.76 × 10^6^
				Oli^−^	1.4	1.8^c^	1.80 × 10^9^	0.0032	5.76 × 10^6^	100	
		FHT	1OH	Oli	18.8	2476.9	2.70 × 10^2^	0.9968	2.69 × 10^2^	0	
			3OH		18.8	2476.9	2.70 × 10^2^		2.69 × 10^2^	0	
			7CH		19.8	47.3	9.40 × 10^−1^		9.37 × 10^−1^	0	
OliA	PE	FHT	7CH	OliA	21.4	66.9	8.70 × 10^−2^	—	—	100	8.70 × 10^−2^
	W	FHT	7CH	OliA^−^	16.3	33.2	2.50 × 10^2^	0.9977	2.49 × 10^2^	0	1.87 × 10^6^
		SET		OliA	—	—	—	0.0010	∼0	0	
				OliA^−^	—	—	—	0.9977	∼0	0	
				OliA^−2^	1.5	2.0^c^	1.20 × 10^9^	0.0013	1.87 × 10^6^	100	
BHT^63^	PE	—									1.70 × 10^4^
	W	—									2.51 × 10^5^

a Mole fraction.

b *k*_f_ = *f* × *k*_app_.

c The nuclear reorganization energy (*λ*).

### 3.3. Comparison with the available experimental data

The comparison of the theoretical predictions was made by reference to the work of Taslimi *et al.*^69^ which evaluated the antioxidant activity of Oli relative to several reference antioxidants, including BHT, using five methods, namely DPPH˙, ABTS˙^+^, DMPD˙^+^, O_2_˙^−^, and metal chelating. The outcomes for Oli and BHT are summarized in Table 3, and the comparison was made solely for Oli since no prior research has been dedicated to examining the antioxidant characteristics of OliA. The examination of the data in Table 3 highlights two significant observations. Firstly, BHT is more active than Oli in the DPPH˙ assay (IC_50_ 7.61 *vs.* 17.77 μM), which primarily relies on hydrogen atom transfer instead of electron transfer. This agrees with the theoretical assessment that reveals that FHT is not the primary mechanism for Oli. Secondly, Oli is more active than BHT in the ABTS˙^+^ assay (IC_50_ 1.94 *vs.* 2.06 μM). This outcome is also consistent with the theoretical predictions since the ABTS˙^+^ assay is relevant for electron-donating antioxidants. Concerning the other techniques, Oli is more active than BHT in the DMPD˙^+^ and metal chelating assays, whereas BHT is more active than Oli in the O_2_˙^−^ scavenging assay. Overall, the experimental findings indicate that Oli is a promising radical scavenger and more potent than BHT in terms of electron donation, which is consistent with the theoretical predictions.

**Table 3:** Reported experimental data (IC_50_ in μM) of the antiradical activity of Oli and BHT^69^

Assays	IC_50_ in μM	
	Oli	BHT
DPPH˙	17.77	7.61
ABTS˙^+^	1.94	2.06
DMPD˙^+^	19.25	21.65
O_2_˙^−^ scavenging	53.30	31.50
Metal chelating	2.83	5.11

### 3.4. Transition metals sequestration and pro-oxidant behavior of OliA

**Fig. 5:**
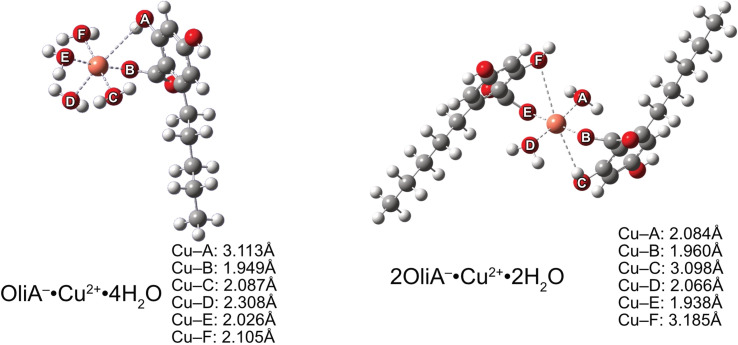
Structures of Cu(ii) mono- and bi-dentate complexes with OliA^−^.

Considering the previously obtained molar fraction values for the possible structures of the tested compounds, it is clear that the neutral and doubly dissociated forms will not significantly affect the type II antioxidant potential. Therefore, the chelation process was studied only for monoanion. Quantum mechanical calculations yielded local ground-state structures for mono- and bidentate complexes formed in a theoretical physiological environment (Fig. 5).

Similar to the results of earlier work,^70,71^ the Jahn–Teller effect is clearly visible in the case of Cu(ii) complexes. One pair of coordinating oxygen atoms of each complex, in mutual axial position, is at a much greater distance from the metal than the others – in the figure these are A and D for neutral compounds and C and F for monoanionic compounds. The distance between the metal and a particular oxygen is particularly large, at as much as 3.185 Å. This is quite a large distance, considering the reported values (see the aforementioned articles).

Moving on to a more detailed analysis of the process, it is necessary to refer to the thermochemical results to determine the stability constants of the complexes formed. With the Gibbs free energy estimated at −9.2 kcal mol^−1^, a chelation constant of 5.39 × 10^6^ M^−1^ s^−1^ was obtained for the monodentate copper complex. For bidentate, it was −12.3 kcal mol^−1^ and 1.10 × 10^9^ M^−1^ s^−1^, respectively. Thus, it was confirmed that copper chelated by the two ligands is more stable, and will be the preferred form under the given conditions. Regardless of these differences, the sequestration process proceeds smoothly.

However, as indicated earlier in the text, the chelation itself does not always prevent the generation of radicals from oxidative damage. Although it can reduce the metal's contribution to reactions that generate reactive oxygen species, it can actually begin to exhibit pro-oxidant potential, transforming physiologically neutral substances into harmful ones *via* an indirect pathway. To explain this, we examined how the complexes formed react with ascorbate and superoxide anion through a single-electron transfer mechanism, thus recognizing pro-oxidation processes that can occur in the body (Table 4).

**Table 4:** Reorganisation energies (*λ*, in kcal mol^−1^), Gibbs free energies (Δ*G*_r_, in kcal mol^−1^), activation energies (Δ*G*^‡^_r_, in kcal mol^−1^), rate constants (*k*, in M^−1^ s^−1^) and apparent rate constants (*k*_app_, in M^−1^ s^−1^) for redox reactions between the studied complexes and physiological reductants

Complex	*λ*	Δ*G*_r_	Δ*G*^‡^_r_	*k*	*k*_app_
**O**_**2**_ **˙** ^**−**^ **/O**_**2**_					
Aquacomplex	31.7	−14.9	2.2	1.45 × 10^11^	3.75 × 10^9^
Monodentate	37.9	−23.4	1.4	6.01 × 10^11^	4.12 × 10^9^
Bidentate	29.0	−20.5	0.6	2.19 × 10^12^	4.47 × 10^9^

**Asc** ^**−**^ **/Asc˙**					
Aquacomplex	26.8	−0.5	6.4	1.18 × 10^8^	1.15 × 10^8^
Monodentate	19.4	−9.0	1.4	5.89 × 10^11^	3.78 ×10^9^
Bidentate	22.8	−27.4	0.2	4.18 × 10^12^	4.35 × 10^9^

The results reveal a number of important relationships regarding the influence of the complexed metal, the metal : ligand molar ratio and the reducing agent. All these data are contrasted with the results obtained for aquacomplexes. Moreover, the results of the aquacomplexes are in agreement with experimental literature data.^72,73^

First, there is the obvious observation that the redox process is extremely fast, with reaction rates at the diffusion limit regardless of the nature of the complex. In the case of the O_2_˙^−^/O_2_ reaction, the monodentate complex has the most negative Δ*G*_r_ value (−23.4 kcal mol^−1^), suggesting that it is the most thermodynamically stable. However, it is actually the bidentate complex that has the highest *k* value (4.47 × 10^9^), indicating that it may have the fastest reaction kinetics. In the case of O_2_˙^−^/O_2_ species, the bidentate complex shows the highest thermodynamic stability and simulatively reacts with ascorbate with the highest rate constant. There is an apparent increase in the pro-oxidation potential of the complexes with the ligand relative to free copper. This indicates that the complexes can undergo chemical reactions more rapidly compared to the aquacomplex and monodentate complexes for both reactions and therefore exhibit the highest pro-oxidation potential.

### 3.5. Inhibitory potential toward ROS-producing enzymes

As mentioned above, antioxidant action can also be exerted through the inhibition of ROS-producing enzymes, thereby reducing OS and limiting ROS production. In this section, the inhibitory potential of Oli and OliA towards ROS-producing enzymes, including myeloperoxidase (MP), cytochrome P450 (CP450), lipoxygenase (LOX), and xanthine oxidase (XO), was assessed using molecular docking studies.

MP, CP450, LOX, and XO are involved in OS through the generation of ROS and other oxidizing agents. MP is a heme enzyme present in neutrophils that produces hypochlorous acid, a potent oxidant that can damage cellular components. CP450 is a family of enzymes that play a crucial role in drug metabolism, but they can also generate ROS during the catalytic cycle. LOX is an enzyme that catalyzes the oxidation of polyunsaturated fatty acids to form lipid hydroperoxides, which can lead to membrane damage and inflammation. XO generates ROS during the conversion of hypoxanthine to xanthine and uric acid. Even though these enzymes are crucial to cell function, their inhibition may reduce OS by limiting the generation of ROS. However, it should be noted that excessive inhibition of these enzymes can alter the proper functioning of cells and thus induce undesirable effects.

To determine the best docking parameters, molecular docking protocols were first validated by redocking co-crystallized ligands with target enzymes. The suitability of each docked ligand's position was evaluated based on binding affinity and RMSD values, which are expected to be less than 2.0 Å according to previous studies.^74^ As shown in Fig. S1 in ESI,† the experimental and docked structures overlapped well, indicating low RMSD values (<1.73 Å). The same protocols were then employed to perform molecular docking simulations and investigate the interactions between the target enzymes' binding sites and the compounds Oli and OliA.

**Fig. 6:**
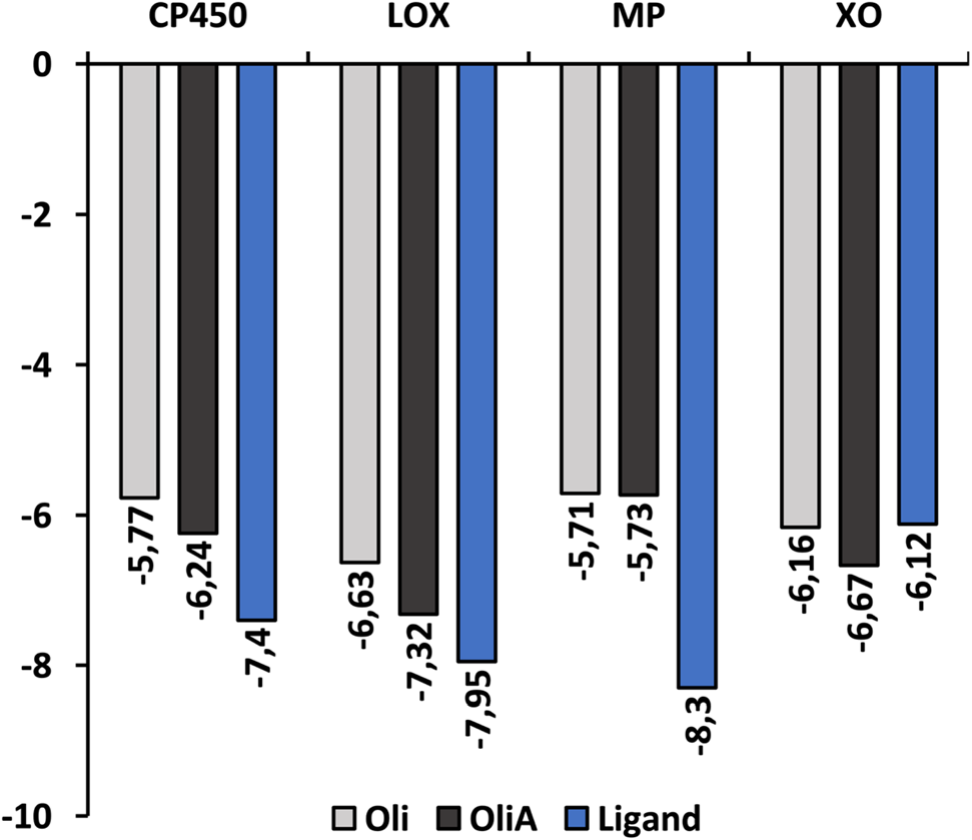
The binding affinity of Oli, OliA, and control ligands with target enzymes.

Fig. 6 shows the binding affinity of Oli and OliA to the target enzymes, along with several control ligands for MP, CP450, LOX, and XO (7-(benzyloxy)-1*H*-[1,2,3]triazolo[4,5-*d*]pyrimidin-5-amine (synthesized inhibitor), *S*-warfarin, (−)-epigallocatechin gallate, and quercetin, respectively). The data indicate that both Oli and OliA exhibit negative binding affinities for all enzymes, with values ranging from −5.71 to −7.32 kcal mol^−1^. These results suggest that Oli and OliA can interact effectively with the active site of the studied enzymes, and therefore may have potential as inhibitors. Interestingly, the binding affinity of OliA was slightly lower than that of Oli and even the control ligand in the case of XO, indicating that OliA may be a better inhibitor than Oli.

**Fig. 7:**
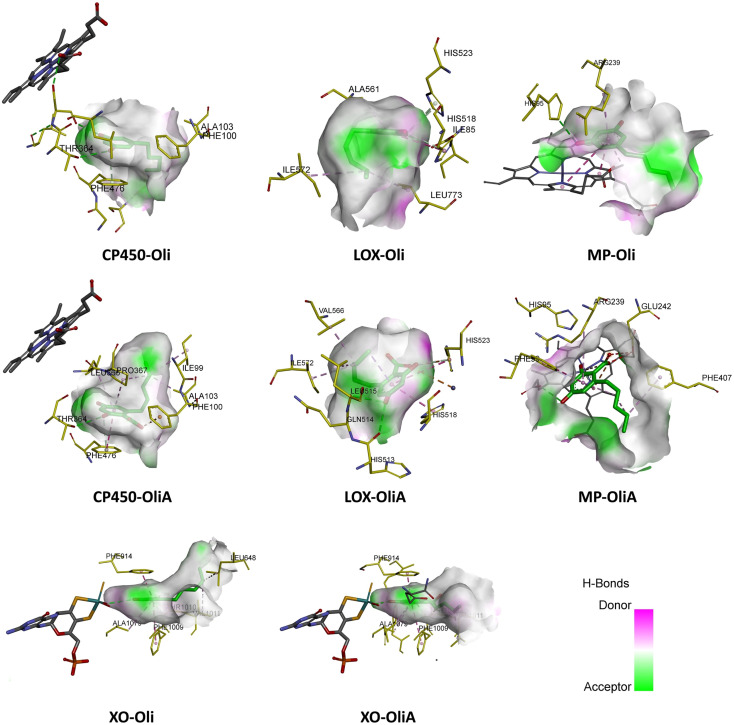
Best docking pose of Oli and OliA in the active site of the target enzymes (myeloperoxidase (MP), cytochrome P450 (CP450), lipoxygenase (LOX), and xanthine oxidase (XO)).

**Fig. 8:**
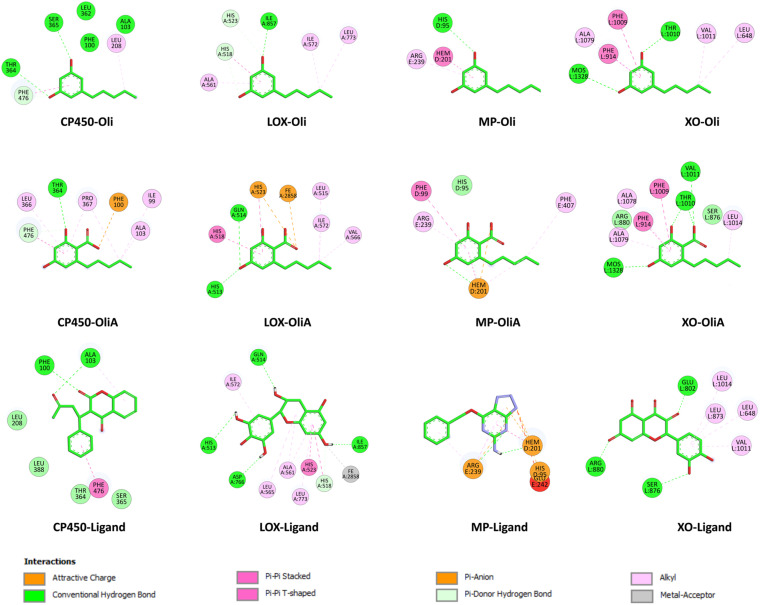
2D representation of the interactions of Oli, OliA, and control ligands with target enzymes (myeloperoxidase (MP), cytochrome P450 (CP450), lipoxygenase (LOX), and xanthine oxidase (XO)).

The interaction modes as well as the 2D representation of the favorable interactions are shown in Fig. 7 and 8, respectively. The results indicate that Oli and OliA exhibit favorable interactions with important amino acid residues in the active sites of the studied enzymes. The 2D representations of the interactions reveal that these compounds share common amino acid residues with the reference ligand in each enzyme, indicating their potential as inhibitors.

In the case of CP450, Oli, OliA, and the reference ligand share favorable interactions with PHE476, while OliA and the reference ligand also interact with PHE100 and ALA103. These residues are reported to play a key role in the catalytic activity of the enzyme.^75^ The representation in Fig. 7 shows that all three molecules are located in the same region near the HEM catalytic center, suggesting their potential to inhibit this enzyme.

Similarly, in the case of LOX, OliA and the reference ligand share almost the same amino acid residues, including ILE572, HIS523, GLN514, HIS518, and HIS513. Oli shares ILE572 and HIS518 with them. These amino acids are involved in the interaction between the enzyme and the native ligand, as described in the literature.^76^ The representation in Fig. 7 shows that all three molecules are located in the same region in the active site of the enzyme, suggesting their potential to inhibit LOX.

Regarding the MP enzyme, all three molecules share favorable interactions with the catalytic center HEM201 and the residue ARG239. Oli forms a favorable interaction with HIS95, similar to the reference ligand. These results are in good agreement with reported studies,^77^ indicating the potential of Oli and OliA to inhibit MP.

Finally, the interaction mode of Oli and OliA with XO is similar to that of the reference ligand. They share favorable interactions with VAL1011 and LEU648 and form favorable interactions with the catalytic center MOS1323, and other important amino acids such as ALA1079, PHE914, PHE109, and THR110.^78^OliA interacts also with the key amino acid ARG880.^78,79^ This suggests that Oli and OliA may have the potential to inhibit XO.

On the basis of these results, it can be seen that Oli and OliA have a good affinity for the studied enzymes by forming stable complexes and interacting with important amino acid residues. These compounds may therefore be good inhibitors of the main ROS-producing enzymes, which may enhance their antioxidant activity at the cellular level. It is crucial to highlight that during the examination of the structure–activity relationship, it becomes evident that the active sites of the enzymes exhibit significant interactions with the carboxylic group of OliA. This implies that the bioactivity of these molecules could be largely influenced by the carboxylic moiety, which may play a critical role in determining their effectiveness.

## 4. Conclusion

The capacity of Oli and OliA as representative alkylresorcinols to scavenge HO˙ and HOO˙ radicals and to inhibit ROS-producing enzymes has been assessed by DFT calculations and molecular docking investigations. The results demonstrate that Oli and OliA are excellent scavengers of HO˙ radical in both polar and lipid media, acting exclusively *via* the FHT mechanism. They are also potent scavengers of HOO˙ radical in water through the SET mechanism, outperforming the common antioxidant BHT. In lipid media, they exhibit moderate HOO˙ radical scavenging activity *via* the FHT mechanism. The OliA anion, which was the only significant transition metal chelator, was able to effectively chelate Cu^2+^ ions, but with a noticeable increase in pro-oxidation potential observed in contrast to the aquacomplexes. Additionally, Oli and OliA display a good affinity for ROS-producing enzymes, including MP, CP450, LOX, and XO, indicating their potential as natural antioxidants. Upon analyzing the structure–activity relationship, it is evident that the radical scavenging action of OliA is not significantly affected by the carboxylic acid group. Nevertheless, it is highly likely that this moiety plays a crucial role in the interaction between the molecule and enzymes as well as in metal chelation. Overall, these findings suggest that Oli and OliA are potent antioxidants and could be utilized for the development of effective antioxidant therapies.

## Conflicts of interest

There are no conflicts to declare.

## Supplementary Material

RA-013-D3RA05967G-s001
