# Supplementary material for: Computational assessment of the primary and secondary antioxidant potential of alkylresorcinols in physiological media
Source: RSC Adv. 2023 Oct 9;13(42):29463–76. doi: 10.1039/d3ra05967g (PMC10561184; doi:10.1039/d3ra05967g)
Supplement: RA-013-D3RA05967G-s001 [file RA-013-D3RA05967G-s001.pdf]

Electronic Supplementary Material (ESI) for RSC Advances.
This journal is © The Royal Society of Chemistry 2023

Computational Assessment of the Primary and Secondary Antioxidant Potential of Alkylresorcinols in Physiological Media

Houssem Boulebd[1,*] & Maciej Spiegel[2]

[1]Department of Chemistry, Faculty of Exact Science, University of Constantine 1, Constantine, 25000, Algeria
[2]Department of Pharmacognosy and Herbal Medicines, Faculty of Pharmacy, Wroclaw Medical University, Borowska 211A, 50-556 Wroclaw, Poland
*Corresponding authors: boulebd.houssem@umc.edu.dz (H.B.)

Table of Contents	Page
**Table S1.** calculated BDE (in kcal/mol) and ΔG (in kcal/mol) of the reaction of Oli and OliA with $HOO^•$ and $HO^•$ radicals in the gas phase computed at M06–2X/6-311++G(d,p) level.	S2
**Table S2.** $\Delta G^{\neq}$ (kcal/mol), κ, *k* ($M^{-1}s^{-1}$), and Γ (%) of the reaction of Oli and OliA with $HO^•$ radical *via* RAF mechanism under physiological conditions, computed at M06-2X/6-311++G(d,p) level.	S3
**Figure S1.** Parameters obtained in the validation of docking protocols for Myeloperoxidase (MP), cytochrome P450 (CP450), lipoxygenase (LOX), and xanthine oxidase (XO) enzymes**.**	S4
**Table S3.** The cartesian coordinates and energies of selected TSs of the reactions of Oli and OliA with $HOO^•$ and $HO^•$ radicals computed at M06-2X/6-311++G(d,p) level.	S5-S12

Table S1. calculated BDE (in kcal/mol) and ∆G (in kcal/mol) of the reaction of Oli and OliA with $HOO^{•}$ and $HO^{•}$ radicals in the gas phase computed at M06–2X/6-311++G(d,p) level.

FHT							RAF				
Position	BDE		ΔG° ($HOO^{•}$)		ΔG° ($HO^{•}$)		Position	ΔG° ($HOO^{•}$)		ΔG° ($HO^{•}$)	
	Oli	OliA	Oli	OliA	Oli	OliA		Oli	OliA	Oli	OliA
1OH	86.1	96.5	2.5	10.6	-28.9	-20.8	C1	15.6	21.7	-11.5	-6.5
3OH	86.1	91.2	2.5	7.2	-28.9	-24.2	C2	14.5	17.3	-10.4	-10.6
7CH	83.7	85.3	1.3	1.1	-30.1	-30.3	C3	15.6	19.9	-11.5	-6.5
							C4	12.2	14.9	-10.6	-8.2
							C5	12.2	17.5	-10.6	-11.3
							C6	18.6	22.7	-8.3	-8.9

Table S2. ΔG≠ (kcal/mol), κ, *k* ($M^{-1}s^{-1}$), and Γ (%) of the reaction of Oli and OliA with $HO^{\bullet}$ radical *via* RAF mechanism under physiological conditions, computed at M06-2X/6-311++G(d,p) level.

Comp.	Sol.	Position	State	ΔG≠	κ	k_{app}	f^{a}	k_f^{b}	Γ	$\sum k_{RAF}$
**Oli**	PE	C1	Oli	9.0	1.1	2.00×10^{6}	-	-	3.4	5.81×10^{7}
		C2		6.2	0.2	2.80×10^{7}			48.2	
		C3		9.0	1.1	2.00×10^{6}			3.4	
		C4		5.3	0.0	1.30×10^{7}			22.4	
		C5		10.6	1.2	1.20×10^{5}			0.2	
		C6		5.3	0.0	1.30×10^{7}			22.4	
	W	C1		6.1	0.1	2.00×10^{7}	0.9968	1.99×10^{7}	46.0	4.48×10^{7}
		C2		2.1	0.0	1.60×10^{5}		1.59×10^{5}	0.4	
		C3		6.1	0.1	2.00×10^{7}		1.99×10^{7}	46.0	
		C4		2.2	0.0	1.20×10^{5}		1.20×10^{5}	0.3	
		C5		8.6	1.1	3.10×10^{6}		3.09×10^{6}	7.1	
		C6		2.2	0.0	1.20×10^{5}		1.20×10^{5}	0.3	
**OliA**	PE	C1	OliA	13.3	1.3	1.60×10^{3}	-	-	0.0	1.04×10^{8}
		C2		6.7	0.6	4.30×10^{7}			41.3	
		C3		12.4	1.3	6.90×10^{3}			0.0	
		C4		4.5	0.0	4.10×10^{7}			39.4	
		C5		11.2	1.2	4.40×10^{4}			0.0	
		C6		7.5	1.0	2.00×10^{7}			19.2	
	W	C1	$OliA^{-}$	8.4	1.1	4.70×10^{6}	0.9977	4.69×10^{6}	28.4	1.65×10^{7}
		C2		0.7	0.0	9.20×10^{5}		9.18×10^{5}	5.6	
		C3		8.2	1.1	7.20×10^{6}		7.18×10^{6}	43.5	
		C4		7.2	0.0	2.60×10^{4}		2.59×10^{4}	0.2	
		C5		8.6	1.1	3.60×10^{6}		3.59×10^{6}	21.8	
		C6		3.0	0.0	9.40×10^{4}		9.38×10^{4}	0.6	

[a]Mole fraction; [b]$k_f = f.k_{app}$

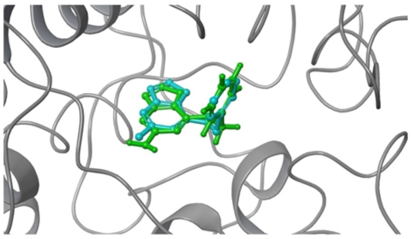

Enzyme : myeloperoxidase (MP, PDB: 5WDJ)
Ligand : 7-(benzyloxy)-1H-[1,2,3]triazolo[4,5-d]pyrimidin-5-amine
BA : -8.30 kcal/mol
RMSD : 0.27 A

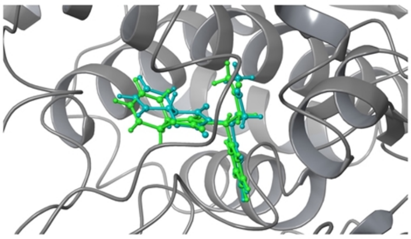

Enzyme : cytochrome P450 (CP450, PDB: 1OG5)
Ligand : S-warfarin
BA : -7.40 kcal/mol
RMSD : 0.65 A

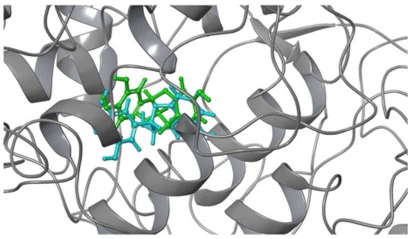

Enzyme : lipoxygenase (LOX, PDB: 1JNQ)
Ligand : (-)-epigallocatechin gallate
BA : -7.95 kcal/mol
RMSD : 1.73 A

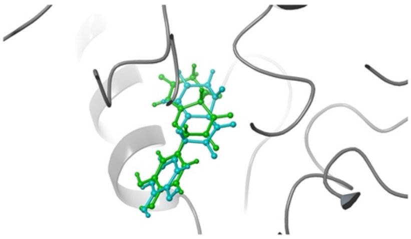

Enzyme : xanthine oxidase (XO, PDB: 3NVY)
Ligand : quercetin
BA : -6.12 kcal/mol
RMSD : 1.73 A

Figure S1. Parameters obtained in the validation of docking protocols for Myeloperoxidase (MP), cytochrome P450 (CP450), lipoxygenase (LOX), and xanthine oxidase (XO) enzymes**.**

Table S3. The cartesian coordinates and energies of selected TSs of the reactions of Oli and OliA with HOO• and HO• radicals computed at M06-2X/6-311++G(d,p) level.

Oli-7C…H…OH (PE)				
Cartesian Coordinates				Frequency and Energy
C	3.41680000	0.13370000	-0.43750000	Zero-point Energy Correction = 0.258664 Hartree
C	2.51750000	1.19850000	-0.47150000	Thermal Correction to Energy = 0.274598 Hartree
C	1.18460000	1.02030000	-0.12180000	Thermal Correction to Enthalpy = 0.275543 Hartree
C	0.73820000	-0.24370000	0.27870000	Thermal Correction to Free Energy = 0.213338
C	1.62900000	-1.31150000	0.32650000	Hartree
C	2.96160000	-1.11630000	-0.03770000	EE + Zero-point Energy = -654.6576 Hartree
O	3.78550000	-2.19590000	0.02020000	EE + Thermal Energy Correction = -654.64167
O	3.01300000	2.40380000	-0.85830000	Hartree
C	-0.70090000	-0.44360000	0.64950000	EE + Thermal Enthalpy Correction = -654.64073
C	-1.70370000	-0.16800000	-0.46580000	Hartree
C	-3.15050000	-0.33670000	-0.01150000	EE + Thermal Free Energy Correction = -654.70293
C	-4.15950000	-0.12260000	-1.13750000	Hartree
C	-5.60140000	-0.27510000	-0.66250000	
H	4.45270000	0.29690000	-0.71540000	
H	0.50010000	1.86330000	-0.14340000	
H	1.30430000	-2.29670000	0.64020000	
H	4.67500000	-1.94280000	-0.25170000	
H	2.31210000	3.06500000	-0.83110000	
H	-0.85420000	-1.43810000	1.07660000	
H	-1.55500000	0.84460000	-0.85520000	
H	-1.49360000	-0.85630000	-1.29420000	
H	-3.28590000	-1.34050000	0.40980000	
H	-3.35910000	0.37540000	0.79660000	
H	-4.01530000	0.87570000	-1.56500000	
H	-3.95850000	-0.83840000	-1.94210000	
H	-6.31180000	-0.12260000	-1.47830000	
H	-5.77300000	-1.27430000	-0.25170000	
H	-5.83180000	0.45140000	0.12220000	
H	-0.92150000	0.29620000	1.49910000	
O	-1.36870000	1.21310000	2.63690000	
H	-2.11220000	0.66280000	2.93990000	
Oli-7C…H…OH (W)				
Cartesian Coordinates				Frequency and Energy
C	3.41912600	0.37757000	-0.25947300	Zero-point Energy Correction = 0.257737 Hartree
C	2.48288700	1.29070700	0.22152800	Thermal Correction to Energy = 0.273792 Hartree
C	1.15306000	0.93185900	0.40347900	Thermal Correction to Enthalpy = 0.274736 Hartree
C	0.74451900	-0.37240200	0.10339200	Thermal Correction to Free Energy = 0.211828
C	1.67131900	-1.29862200	-0.36990900	Hartree

C	2.99902800	-0.91335100	-0.55141200	EE + Zero-point Energy = -654.66323 Hartree
O	3.86895600	-1.86062100	-1.02449500	EE + Thermal Energy Correction = -654.64717 Hartree
O	2.94047600	2.55124900	0.50543700	EE + Thermal Enthalpy Correction = -654.64623 Hartree
C	-0.68893200	-0.76822500	0.29400900	EE + Thermal Free Energy Correction = -654.70914 Hartree
C	-1.69943600	0.05732300	-0.49306900	
C	-3.14011500	-0.36031900	-0.21798100	
C	-4.15396400	0.44308500	-1.02806500	
C	-5.59263800	0.03599600	-0.72533200	
H	4.45313300	0.67434500	-0.39605200	
H	0.44799200	1.66213900	0.78877500	
H	1.37433700	-2.31468700	-0.60312400	
H	4.75672700	-1.48645800	-1.09032000	
H	2.21435500	3.09247900	0.84029900	
H	-0.82450900	-1.83697800	0.11006700	
H	-1.57160300	1.11920200	-0.25820100	
H	-1.48047700	-0.05851100	-1.56203800	
H	-3.25950800	-1.42721400	-0.44106000	
H	-3.35429900	-0.23735200	0.85092500	
H	-4.01896500	1.50912100	-0.81554500	
H	-3.94808200	0.30838900	-2.09541600	
H	-6.30627100	0.61597300	-1.31495200	
H	-5.75288400	-1.02228200	-0.94965200	
H	-5.82553300	0.19175300	0.33183700	
H	-0.91883000	-0.61966500	1.40383300	
O	-1.27372100	-0.20839600	2.86568200	
H	-2.20575300	-0.48514000	2.80984300	
Oli-7C…H…OOH (P)				
Cartesian Coordinates				Frequency and Energy
C	3.57500500	0.40819700	-0.21487800	Zero-point Energy Correction = 0.262357 Hartree
C	2.57540400	1.36807700	-0.06974800	Thermal Correction to Energy = 0.278246 Hartree
C	1.23138200	1.01384200	-0.08568800	Thermal Correction to Enthalpy = 0.27919 Hartree
C	0.87196000	-0.33098900	-0.25164600	Thermal Correction to Free Energy = 0.217764 Hartree
C	1.86954200	-1.30038600	-0.39166100	EE + Zero-point Energy = -729.80102 Hartree
C	3.20892200	-0.92449400	-0.37440400	EE + Thermal Energy Correction = -729.78513 Hartree
O	4.13447000	-1.90840100	-0.52135800	EE + Thermal Enthalpy Correction = -729.78418 Hartree
O	2.98290400	2.65553500	0.08477400	EE + Thermal Free Energy Correction = -729.84561 Hartree
C	-0.54359500	-0.75272200	-0.24235700	
C	-1.61886800	0.22957200	-0.64681900	
C	-3.01329400	-0.39436200	-0.63719000	
C	-4.11938000	0.62737000	-0.88853100	
C	-5.50815100	-0.00361200	-0.89395000	
H	4.61665500	0.71153700	-0.20120000	
H	0.47458600	1.78144300	0.03516400	
H	1.61393500	-2.34585800	-0.51857200	
H	5.02077400	-1.53043600	-0.49494000	
H	2.21485300	3.22561900	0.20417200	
H	-0.68669200	-1.75138700	-0.66237500	

H -1.60518400 1.09633100 0.02399300<br>H -1.39410300 0.61683500 -1.65004700<br>H -3.06387300 -1.18359300 -1.39628600<br>H -3.18745700 -0.89279200 0.32581700<br>H -4.06947800 1.40638600 -0.11893600<br>H -3.93688300 1.12680500 -1.84644000<br>H -6.28691600 0.74390000 -1.06223000<br>H -5.59179100 -0.75758200 -1.68174700<br>H -5.71742600 -0.49614800 0.06018000<br>H -0.80106000 -0.97219700 0.98766100<br>O -1.20397400 -1.03879600 2.19017100<br>O -1.68111500 0.21899600 2.48136200<br>H -2.63178200 0.15950400 2.29742400	
Oli-7C...H...OOH (W)	
Cartesian Coordinates	Frequency and Energy
C 3.55144700 0.42209300 -0.26401700<br>C 2.54414900 1.37467900 -0.13755300<br>C 1.20169900 1.01375200 -0.14513200<br>C 0.85182600 -0.33541500 -0.29034300<br>C 1.85654200 -1.30195400 -0.41502500<br>C 3.19163900 -0.91472300 -0.40336900<br>O 4.13573900 -1.89819700 -0.53819000<br>O 2.93998300 2.67981600 -0.00770900<br>C -0.56195800 -0.76674400 -0.27100600<br>C -1.64483800 0.21696900 -0.64821800<br>C -3.04142000 -0.39530700 -0.57703900<br>C -4.14194600 0.62802400 -0.84159700<br>C -5.53530600 0.01003900 -0.78839700<br>H 4.59338400 0.72303000 -0.25922600<br>H 0.44630300 1.78484300 -0.04373500<br>H 1.60417400 -2.34995200 -0.52939700<br>H 5.01947100 -1.50911600 -0.53338300<br>H 2.16280100 3.24782700 0.06936700<br>H -0.70365100 -1.76087700 -0.70198800<br>H -1.60283800 1.09361600 0.00817900<br>H -1.45177200 0.58696600 -1.66453400<br>H -3.11840700 -1.21490800 -1.30082300<br>H -3.19298600 -0.83977600 0.41486300<br>H -4.06404500 1.43128900 -0.10051700<br>H -3.97843300 1.08935900 -1.82170600<br>H -6.31280400 0.75755800 -0.96174300<br>H -5.64440700 -0.77035300 -1.54672400<br>H -5.71964000 -0.44631000 0.18839200<br>H -0.79152300 -1.00099000 0.94242400<br>O -1.13927800 -1.02456800 2.19153900<br>O -1.49026700 0.27136400 2.48446800<br>H -2.43712600 0.32666600 2.27449900	Zero-point Energy Correction = 0.262795 Hartree<br>Thermal Correction to Energy = 0.279264 Hartree<br>Thermal Correction to Enthalpy = 0.280208 Hartree<br>Thermal Correction to Free Energy = 0.217232 Hartree<br>EE + Zero-point Energy = -729.80535 Hartree<br>EE + Thermal Energy Correction = -729.78888 Hartree<br>EE + Thermal Enthalpy Correction = -729.78793 Hartree<br>EE + Thermal Free Energy Correction = -729.85091 Hartree

Oli-1O…H…OOH (P)				
Cartesian Coordinates				Frequency and Energy
C	2.54709700	1.13392800	-0.64047900	Thermal Correction to Energy = 0.278328 Hartree
C	1.69089800	1.98681100	0.03028100	Thermal Correction to Enthalpy = 0.279273 Hartree
C	0.46857600	1.51813100	0.53376200	Thermal Correction to Free Energy = 0.217974 Hartree
C	0.08045700	0.18514600	0.36212500	
C	0.92430500	-0.67360700	-0.32646500	EE + Zero-point Energy = -729.80574 Hartree
C	2.16412600	-0.20717200	-0.83510100	EE + Thermal Energy Correction = -729.79002 Hartree
O	2.95272600	-1.02430900	-1.49890300	
O	2.08185200	3.27546000	0.18994200	EE + Thermal Enthalpy Correction = -729.78908 Hartree
C	-1.26437400	-0.27485000	0.86276700	
C	-2.38069500	0.02219000	-0.14660000	EE + Thermal Free Energy Correction = -729.85038 Hartree
C	-3.75510200	-0.40321700	0.36158100	
C	-4.87220300	-0.13194500	-0.64356300	
C	-6.24351800	-0.54644200	-0.11849400	
H	3.50003400	1.48263700	-1.01880700	
H	-0.18611200	2.20153000	1.06964500	
H	0.65029300	-1.70991900	-0.50164400	
H	1.41254700	3.76695500	0.68002300	
H	-1.23345800	-1.35010800	1.06111200	
H	-1.49167300	0.22535900	1.80935900	
H	-2.38671500	1.09529000	-0.37053300	
H	-2.15701100	-0.49279500	-1.08785300	
H	-3.73895800	-1.47309500	0.60343300	
H	-3.97512100	0.12386500	1.29824600	
H	-4.88026100	0.93474700	-0.89374600	
H	-4.65566200	-0.66798000	-1.57408800	
H	-7.02870500	-0.35788500	-0.85450100	
H	-6.26214200	-1.61286400	0.12435400	
H	-6.49739800	0.00627200	0.79062800	
H	3.39724000	-1.77300200	-0.76933300	
O	3.76692100	-2.38966600	0.23511500	
O	3.31643900	-1.62202200	1.26411100	
H	2.43219900	-1.96855800	1.47270200	

Oli-1O…H…OOH (W)				
Cartesian Coordinates				Frequency and Energy
C	3.03406600	-0.52413200	-0.13745000	Zero-point Energy Correction = 0.262672 Hartree
C	2.35968900	-1.69863600	-0.39657400	Thermal Correction to Energy = 0.278955 Hartree
C	1.02052300	-1.87004500	0.00476800	Thermal Correction to Enthalpy = 0.279899 Hartree
C	0.34781900	-0.86525900	0.69494500	Thermal Correction to Free Energy = 0.217466 Hartree
C	1.02639800	0.31359900	0.98983300	
C	2.36721900	0.49298600	0.57055000	EE + Zero-point Energy = -729.80726 Hartree
O	2.99838700	1.62426600	0.85115800	EE + Thermal Energy Correction = -729.79097 Hartree
O	3.02306200	-2.68450800	-1.06317100	
C	-1.10740100	-1.00872000	1.05097300	EE + Thermal Enthalpy Correction = -729.79003 Hartree
C	-1.99833400	-0.29356200	0.02790900	
C	-3.48011200	-0.40000500	0.37229500	EE + Thermal Free Energy Correction = -729.85246

C	-4.37888200	0.30566400	-0.63952500	Hartree
C	-5.85742600	0.19307900	-0.28056500	
H	4.05910200	-0.38275500	-0.45681900	
H	0.51599900	-2.80199500	-0.23388400	
H	0.54199600	1.11576600	1.53883600	
H	2.44367700	-3.44762000	-1.18471700	
H	-1.37482500	-2.06800500	1.09250500	
H	-1.28313600	-0.58073600	2.04244800	
H	-1.70519700	0.76267600	-0.02471100	
H	-1.81714900	-0.72182500	-0.96484400	
H	-3.76312400	-1.45805700	0.43153000	
H	-3.65115200	0.02621900	1.36832100	
H	-4.09239600	1.36135400	-0.69940000	
H	-4.20670100	-0.12236600	-1.63310600	
H	-6.48859900	0.70362300	-1.01147500	
H	-6.16902100	-0.85461700	-0.24170300	
H	-6.05326600	0.63578700	0.70018300	
H	2.47080100	2.46353800	0.33644100	
O	1.79026500	3.18524500	-0.45699400	
O	0.98775700	2.28385600	-1.08726500	
H	0.17585600	2.22833000	-0.55238000	
OliA-7C…H…OH (P)				
Cartesian Coordinates				Frequency and Energy
C	-2.96788800	1.55646500	-0.01610900	Zero-point Energy Correction = 0.274307 Hartree
C	-1.80890900	2.28241800	0.17399700	Thermal Correction to Energy = 0.292435 Hartree
C	-0.55535000	1.65720500	0.15703200	Thermal Correction to Enthalpy = 0.293379 Hartree
C	-0.44093100	0.28912100	-0.02381500	Thermal Correction to Free Energy = 0.226322 Hartree
C	-1.62706300	-0.48512300	-0.18671900	EE + Zero-point Energy = -843.22436 Hartree
C	-2.88203400	0.18155100	-0.21221900	EE + Thermal Energy Correction = -843.20623 Hartree
O	-4.04298900	-0.45420100	-0.40595300	EE + Thermal Enthalpy Correction = -843.20529 Hartree
O	-1.93018200	3.61362000	0.35682500	EE + Thermal Free Energy Correction = -843.27234 Hartree
C	0.93794500	-0.31306200	-0.07409800	
C	2.12304300	0.63607400	-0.17290500	
C	3.43614900	-0.13223900	-0.30542000	
C	4.65735900	0.78246900	-0.35250500	
C	5.96005500	0.00395400	-0.50882900	
C	-1.66776200	-1.94530000	-0.30642500	
O	-2.65068900	-2.58034900	-0.65878800	
O	-0.55203600	-2.60850900	0.03556400	
H	-3.93583300	2.04049300	-0.02517900	
H	0.32986400	2.26968500	0.27489700	
H	-3.85027400	-1.39621200	-0.58775400	
H	-1.06035100	4.01119500	0.48532500	
H	1.00080900	-1.07871500	-0.84853400	
H	1.99534400	1.29510500	-1.04081200	
H	2.17005700	1.27746800	0.71339800	
H	3.53380700	-0.82708200	0.53707800	

H 3.40729000 -0.74512500 -1.21440300<br>H 4.54842100 1.48945500 -1.18267900<br>H 4.69286600 1.38098600 0.56438500<br>H 6.82578800 0.66994300 -0.53388400<br>H 6.09875600 -0.69445700 0.32139100<br>H 5.95819200 -0.57782500 -1.43510500<br>H -0.72204500 -3.55105600 -0.12511700<br>H 1.09310000 -0.89149800 0.90561000<br>O 1.22171800 -1.65132200 2.20186000<br>H 0.55994900 -2.30746200 1.92340400	
OliA-7C…H…OH (W)	
Cartesian Coordinates	Frequency and Energy
C -3.20448200 0.77251400 -0.56966100<br>C -2.32183100 1.83347500 -0.46236700<br>C -1.00824700 1.62834200 -0.04561900<br>C -0.55356900 0.34782000 0.26049500<br>C -1.42287300 -0.75951800 0.14031100<br>C -2.75961500 -0.51024600 -0.26177700<br>O -3.65811800 -1.51538100 -0.36319100<br>O -2.79340200 3.07975800 -0.76590900<br>C 0.88306700 0.22934400 0.69524000<br>C 1.87241000 0.09803200 -0.45800600<br>C 3.30948700 -0.05403500 0.02797200<br>C 4.31299800 -0.18181600 -1.11469000<br>C 5.74551300 -0.33896000 -0.61404900<br>C -0.99512300 -2.18686000 0.38245500<br>O -1.93130900 -3.06204700 0.42454800<br>O 0.20838800 -2.47679000 0.50957300<br>H -4.23077100 0.93013900 -0.87813400<br>H -0.34012900 2.47848400 0.05027300<br>H -3.14168800 -2.33514700 -0.04745500<br>H -2.10106900 3.73611800 -0.61574400<br>H 1.02936200 -0.55369600 1.43593500<br>H 1.59064300 -0.77279300 -1.06165500<br>H 1.78585200 0.97671100 -1.10781700<br>H 3.57499100 0.80909500 0.65067000<br>H 3.37737800 -0.93919500 0.67220600<br>H 4.04152900 -1.04275600 -1.73515500<br>H 4.24179800 0.70226300 -1.75773100<br>H 6.45305300 -0.43022000 -1.44130400<br>H 6.04263600 0.52403500 -0.01143800<br>H 5.84224900 -1.23131000 0.01085300<br>H 1.16596700 1.20017500 1.23309400<br>O 1.53749200 2.47836300 2.00117300<br>H 0.61737100 2.64833800 2.27202100	Zero-point Energy Correction = 0.259457 Hartree<br>Thermal Correction to Energy = 0.277374 Hartree<br>Thermal Correction to Enthalpy = 0.278319 Hartree<br>Thermal Correction to Free Energy = 0.211098 Hartree<br>EE + Zero-point Energy = -842.79174 Hartree<br>EE + Thermal Energy Correction = -842.77383 Hartree<br>EE + Thermal Enthalpy Correction = -842.77288 Hartree<br>EE + Thermal Free Energy Correction = -842.8401 Hartree
OliA-7C…H…OOH (P)	
Cartesian Coordinates	Frequency and Energy

C	-2.96026600	1.60347500	-0.11213400	Zero-point Energy Correction = 0.278523 Hartree
C	-1.81431800	2.31535000	0.19388500	Thermal Correction to Energy = 0.297581 Hartree
C	-0.55618200	1.70647400	0.15858700	Thermal Correction to Enthalpy = 0.298525 Hartree
C	-0.42290000	0.35650000	-0.14841800	Thermal Correction to Free Energy = 0.229232 Hartree
C	-1.60191700	-0.40657700	-0.40508400	
C	-2.85609200	0.25251700	-0.43000800	EE + Zero-point Energy = -918.36697 Hartree
O	-4.00251200	-0.37171900	-0.73026900	EE + Thermal Energy Correction = -918.34791 Hartree
O	-1.95897000	3.62274100	0.49559500	
C	0.93723000	-0.21425100	-0.25167100	EE + Thermal Enthalpy Correction = -918.34697 Hartree
C	2.13544000	0.71043700	-0.30799500	
C	3.43756700	-0.06988300	-0.47837500	EE + Thermal Free Energy Correction = -918.41626 Hartree
C	4.66954900	0.83091800	-0.49898100	
C	5.96121400	0.04273400	-0.69284000	
C	-1.62762500	-1.86008900	-0.59569900	
O	-2.54828400	-2.47270400	-1.11186800	
O	-0.57778600	-2.53034500	-0.09566000	
H	-3.93048000	2.08320400	-0.11565900	
H	0.32160000	2.31172300	0.35019000	
H	-3.79512500	-1.28383600	-1.00979900	
H	-1.10079200	4.01135500	0.70384300	
H	1.00977100	-1.03852100	-0.95959100	
H	2.01744600	1.41340900	-1.14398600	
H	2.19403500	1.31327300	0.60455300	
H	3.53558100	-0.79769000	0.33614800	
H	3.39370400	-0.64731400	-1.40948400	
H	4.56298100	1.56924600	-1.30163400	
H	4.71815400	1.39427900	0.43927000	
H	6.83477600	0.69877500	-0.69957600	
H	6.09676000	-0.68711200	0.11046100	
H	5.94611700	-0.50462600	-1.63968900	
H	-0.70001800	-3.46948500	-0.30958300	
H	1.10243500	-0.84526300	0.85262900	
O	1.20753300	-1.35620800	1.98857200	
O	-0.05867800	-1.27610200	2.51145200	
H	-0.50758300	-2.05236100	2.14173900	
OliA-7C…H…OOH (W)				
Cartesian Coordinates				Frequency and Energy
C	-2.95801800	1.60043600	-0.09402800	Zero-point Energy Correction = 0.263842 Hartree
C	-1.82085500	2.33550100	0.20472600	Thermal Correction to Energy = 0.282625 Hartree
C	-0.55991300	1.74707300	0.18195200	Thermal Correction to Enthalpy = 0.283569 Hartree
C	-0.41907100	0.38530300	-0.10580300	Thermal Correction to Free Energy = 0.21504 Hartree
C	-1.57479200	-0.39444200	-0.35965700	
C	-2.82457900	0.24994500	-0.40021000	EE + Zero-point Energy = -917.939 Hartree
O	-3.96470600	-0.41999700	-0.72187800	EE + Thermal Energy Correction = -917.92021 Hartree
O	-1.99167600	3.66167100	0.48790900	
C	0.94054000	-0.19277200	-0.19451800	EE + Thermal Enthalpy Correction = -917.91927 Hartree
C	2.13776300	0.72412000	-0.33294000	

C	3.44207700	-0.06373100	-0.42960700	EE + Thermal Free Energy Correction = -917.9878 Hartree
C	4.66948400	0.83765600	-0.52317200	
C	5.96652000	0.04209600	-0.63093300	
C	-1.55177800	-1.89354900	-0.54779800	
O	-2.39229900	-2.38207500	-1.35449900	
O	-0.75272900	-2.58281600	0.13633100	
H	-3.93474700	2.06827800	-0.11006700	
H	0.30784400	2.36904500	0.36587800	
H	-3.68113300	-1.29347600	-1.07638200	
H	-1.13613200	4.07236600	0.66733700	
H	0.98914100	-1.05722000	-0.85867800	
H	2.01629100	1.34703400	-1.22966400	
H	2.19484900	1.40911600	0.51919400	
H	3.53584000	-0.71486800	0.44830300	
H	3.40489500	-0.72185400	-1.30548200	
H	4.56699900	1.49687400	-1.39218000	
H	4.70492600	1.48622400	0.35879000	
H	6.83727400	0.69925900	-0.68777500	
H	6.09476800	-0.61088600	0.23702700	
H	5.96232100	-0.58864400	-1.52436300	
H	1.12703800	-0.76807300	0.91361700	
O	1.18021100	-1.38331500	2.03849200	
O	-0.14024700	-1.43495500	2.42920200	
H	-0.54050200	-2.00023700	1.71838600	
